# Positive Attitude Towards Personal Drug Selection among Undergraduate Medical Students of a Medical College

**DOI:** 10.31729/jnma.8434

**Published:** 2024-02-29

**Authors:** Anjan Khadka, Sanjog Gurung, Sujata Ghimire, Suvam Lahera, Toslima Mabuhang, Neha Shah

**Affiliations:** 1 Department of Pharmacology, Nepalese Army Institute of Health Sciences, Sanobharyang, Kathmandu, Nepal; 2 Nepalese Army Institute of Health Sciences, Sanobharyang, Kathmandu, Nepal

**Keywords:** *medical students*, *medicine*, *perception*, *prevalence*

## Abstract

**Introduction:**

Personal drug exercise helps in the selection of drugs depending on the criteria-safety, efficacy, suitability and cost. Undergraduate medical students are the future practitioners-in-training and should focus more on rational prescribing. This study aimed to find out the prevalence of positive attitudes towards personal drug selection among undergraduate medical students of a medical college.

**Methods:**

This is a descriptive cross-sectional study conducted among second and third-year medical students after obtaining ethical approval from the Institutional Review Committee. Data was collected from 1 December 2022 to 30 May 2023. A convenience sampling method was used. The point estimate was calculated at a 95% Confidence Interval.

**Results:**

Among 132 medical students, 126 (95.45%) (91.90-99.01, 95% Confidence Interval) of the students showed a positive attitude toward P-drug selection.

**Conclusions:**

The prevalence of positive attitudes towards P-drug selection among medical students was found to be similar to other studies done in similar settings.

## INTRODUCTION

The selection of personal (P) drugs is an important aspect of the pharmacology practical in the medical curriculum of Nepal. The Guide to Good Prescription and the Teachers Guide to Good Prescription provide a good understanding of the concepts of P dug.^1,2^ P drug's practical exercise aids in the prescription of medications based on objective efficacy, safety, cost, convenience/suitability, and unbiased sources of medicine information that is dosage form, dosage schedule and duration of treatment.^2^

P-drug selection exercises help students to make prescribing decisions based on impartial, objective information and the activity of P-drug selection can help to reduce irrational prescribing, which is a worldwide problem.^3-5^ It helps to guide educational interventions for P-drug selection exercises that can improve students' abilities to make well-informed, patientcentred decisions about medication therapy by identifying obstacles and knowledge gaps.^5^

This study aimed to find out the prevalence of positive attitudes towards personal drug selection among undergraduate medical students of a medical college.

## METHODS

This descriptive cross-sectional study was conducted among the second and third-year undergraduate medical students of the Nepalese Army Institute of Health Sciences, Sanobharyang, Kathmandu, Nepal. Data was collected from 1 December 2022 to 30 May 2023. Ethical approval was obtained from the Institutional Review Committee of the same institute (Reference number: 677). The students of second and third-year Bachelor of Medicine, Bachelor of Surgery who gave consent, present in the lecture hall during the questionnaire administration period and who were exposed to P-drugs practical in Pharmacology in their integrated basic medical sciences were included in the study. A convenience sampling method was used. The sample size was calculated by using the following formula:


$$\begin{array}{ll} \text{n} & ={\text{Z}}^{2}\times \frac{\text{p}\times \text{q}}{{\text{e}}^{\text{2}}}\\ & =1.96^{2}\times \frac{0.50\times 0.50}{0.05^{2}}\\ & =385 \end{array}$$

Where,

n = minimum required sample size
Z = 1.96 at 95 % Confidence Interval (CI)
p = prevalence taken as 50% for maximum sample size
q = i-p
e = margin of error, 5%

The sample size was adjusted for a finite population as follows:


$$\begin{array}{ll} \text{n} & =\frac{\text{n}}{1+\frac{\text{n}-1}{\text{N}}}\\ \text{n} & =\frac{\text{385}}{1+\frac{\text{385}-1}{\text{200}}}\\ & =132 \end{array}$$

Where,

n' = adjusted sample size
N = finite population

The calculated minimum required sample size was 132.

The structured self-administered questionnaire from previously validated and published literature consisting of eleven statements was given out in a regular lecture hall during leisure hours after getting permission from the respective year coordinators. Questionnaire had two parts-the first part included demographic-related questions and the second part consisted of statements regarding P-drug. Statements followed five-point Likert-type scale responses: 1-Strongly disagree, 2-Disagree, 3-Uncertain, 4-Agree, and 5-Strongly agree.^5-7^ The attitude was determined based on the argument that a score between 2.5 to 3.4 on the Likert scale represents a neutral attitude, a score less than 2.4 for a negative attitude and a score more than 3.4 to 5 for a positive attitude.^8^

Data were entered and analysed using Microsoft Excel 2007. The point estimate was calculated at a 95% CI.

## RESULTS

Among 132 medical students, 126 (95.45%) (91.89-99.1, 95% CI) showed a positive attitude toward the p-drug selection. A total of 84 (66.67%) were male and 42 (33.33%) were female. A total of 37 (29.37%) of the students agreed that p-drug exercises will have a long-term impact as compared to prescription writing exercises (Table 1).

**Table 1:** Student's perception of P-drug selection exercise (n = 126).

Statements	Strongly agree	Agree	Uncertain	Disagree	Strongly disagree
P-drug concept gives in depth knowledge about the drug, classification to which drug belongs and other drugs in this group.	72 (57.14)	54 (42.86)	2 (1.59)	3 (2.38)	1 (0.79)
While comparing the different drugs in same group the intragroup variability is not highlighted.	11 (8.73)	18 (14.29)	3 (2.38)	55 (43.65)	45 (35.71)
As compared to prescription writing, P-drug concept helps in selecting a drug for a disease rather than for a patient.	17 (13.49)	17 (13.49)	19 (15.08)	40 (31.75)	39 (30.95)
Using CIMS, textbooks and reference books is helpful in selecting P-drugs.	40 (31.75)	38 (30.16)	7 (5.56)	36 (28.57)	11 (8.73)
P-drug exercises will have a long-term impact on your mind as compared to prescription writing exercises.	37 (29.37)	28 (22.22)	28 (22.22)	23 (18.25)	16 (12.70)
P-drug selection gives a better idea of cost of different drug in same group.	59 (46.83)	37 (29.37)	9 (7.14)	10 (7.94)	17 (13.49)
Teaching of P-drug selection is a two-way learning process where students get equal chance to present their view.	56 (44.44)	43 (34.13)	13 (10.32)	14 (11.11)	6 (4.76)
Some difficult topics which are not properly covered in theory are better understood.	30 (23.81)	20 (15.87)	16 (12.70)	44 (34.92)	22 (17.46)
Selection of P-drug is a time-consuming process.	35 (27.78)	32 (25.40)	2 (1.59)	48 (38.10)	15 (11.90)
Comparing efficacy is more difficult than comparing safety while selecting a P-drug.	11 (8.73)	15 (11.90)	11 (8.73)	32 (25.40)	63 (50)
P-drug selection exercises in undergraduate pharmacology practical curriculum are helpful.	41 (32.54)	28 (22.22)	8 (6.35)	40 (31.75)	15 (11.90)

Most of the students 62 (49.21%) used the library to access the books and resources needed for P-drug selection practical exercises (Table 2).

**Table 2:** Mode to access the books and resources needed for the P-drug selection exercise (n = 126).

Books and Resources	n (%)
Library	62 (49.21)
Internet	22 (17.46)
PDF versions of textbooks	21 (16.67)
Practical Lab	18 (14.29)
Textbook	11 (8.73)
Journal	1 (0.79)

## DISCUSSION

In this study among 132 medical students, 126 (95.45%) had a positive attitude on P-drug selection exercises which is similar to another study with a prevalence of 90%.^5^ Another study reported the prevalence of positive attitude on p-drug selection with 74.9%.^9,10^ P-drug practical is dynamically understood and perceived by medical undergraduates and also included in the university final practical exam, hence it is encouraged in our settings.

The P-drug concept aids in choosing a medication for a disease as opposed to a patient because P-drugs are the primary choice for a certain indication. Most of the students of our study also opined the same which is similar to the study conducted in India.^4^ Exploring the knowledge and perception of P-Drug practical among medical undergraduates in Nepal can provide insights into their understanding of rational prescribing principles and guide future interventions for improved prescribing practices.^8^

The majority of students felt that using Current Index of Medical Specialities, textbooks, and reference materials helped them choose a P-drug and most of them concur that, in comparison to prescription writing exercises, P-drug exercises have a longer-lasting effect on their thinking. Similar findings were reported by previous studies.^4,6^ The P-drug concept, which emphasizes rational prescribing and evidence-based medicine, has the potential to have a lasting impact on students' perspectives on and methods for prescribing drugs. P-drugs are selected for their effectiveness, safety, and cost-effectiveness, which encourages students to think about the best course of action for a certain ailment. This logical method of prescribing decreases the likelihood of side effects and helps avoid using medications that aren't necessary. P-drug training frequently emphasizes modifying therapies to meet the specific needs of each patient. Students are more likely to retain and apply this knowledge in their future clinical practice since P-drugs concentrate on critical medicines for particular illnesses, ensuring improved patient care and safety. By comparing the costs of drugs within a therapeutic group, students can gain a better idea of the cost-effectiveness of different treatment options and make informed decisions accordingly.^2,4^

The choice of P-drug was acknowledged by the students as being time-consuming in another study but our study showed a balanced response from the students.^4,6^ In comparison to prescription writing exercises, which primarily involve tailoring a prescription to an individual patient, P-drug exercises emphasize the broader concept of selecting the most appropriate drug for a given condition. Our study findings supported this broader concept which is similar to studies conducted in Nepal and India.^5,6,11^ Regarding the inclusion of P-drug selection exercises in undergraduate Pharmacology practical courses, this study found neutral perception. However, various studies reported that P-drug selection exercises should be incorporated into the curriculum.^4,6,12,13^ The male-to-female ratio in our study was 1.93 which is higher than that of the previous study with a ratio of 1.45.^4^

There are a few limitations in our study. The study was limited to a single medical college, making it difficult to generalize the findings to a larger setting. Hence, the features of the college are not representative, sampling bias may cause the number of positive opinions to be overestimated or underestimated. Furthermore, there might be self-reporting bias. In order to encourage accurate responses, anonymity and confidentiality were emphasized.

## CONCLUSIONS

The prevalence of positive attitudes towards P-drug selection was found to be similar to other studies done in similar settings. Further research involving a broader and diverse sample of medical students across multiple centres should be conducted to enhance the generalizability of findings, and incorporating feedback from students could inform the refinement of P-drug exercises, ensuring they remain an effective and integral component of pharmacology education.
